# Effect of Wall Thickness on Stress–Strain Response and Buckling Behavior of Hollow-Cylinder Rubber Fenders

**DOI:** 10.3390/ma13051170

**Published:** 2020-03-05

**Authors:** Ming-Yuan Shen, Yung-Chuan Chiou, Chung-Ming Tan, Chia-Chin Wu, Wei-Jen Chen

**Affiliations:** 1 Department of Mechanical Engineering, National Chin-Yi University of Technology, Taichung 41170, Taiwan; hbj678@gmail.com; 2 Department of Biomechatronic Engineering, National Chiayi University, Chiayi 60004, Taiwan; 3 Graduate School of Opto-Mechatronics and Materials, WuFeng University, Chiayi 62153, Taiwan; cmtan@wfu.edu.tw; 4 Department of Mechanical and Energy Engineering, National Chiayi University, Chiayi 60004, Taiwan; joechia-chin.wu@mail.ncyu.edu.tw; 5 Department of Aeronautical Engineering, Chaoyang University of Technology, Taichung 413310, Taiwan; dancerbear@cyut.edu.tw

**Keywords:** wall thickness, rubber material, hollow-cylinder buckling fender

## Abstract

In this study, the effect of wall thickness (15–25 mm) on the stress–strain response of hollow-cylinder rubber fenders were investigated by conducting monotonic compression tests. It was found that a progressive increase in lateral bending deformation was observed during monotonic compression. Simultaneously, the extent of the lateral deflection decreased notably with an increasing wall thickness. From the experimental results, the fact is accepted that buckling occurred in the tested fender due to the fact that the ratio of the height to the wall thickness was higher than four in all of the considered cases. Moreover, an s-shape profile appeared in the stress–strain curves, which became clearer as the wall thickness was reduced from 25 to 15 mm. To assess the performance of fenders objectively, an energy-effectiveness index, $C_{ER}$, was introduced to quantify the energy absorption capacity of the fender. From the experimental observations, it was inferred that the contact area of the folded inner surface of the fender produced under compression generated an additional reaction force and affected the shape of the stress–strain curve since the measured load consisted of two reaction forces: one caused by the self-contact area, and the other resulted from the compression-bending deformation that occurred in the side wall of the fender. To examine this assertion, a finite element analysis (FEA) was conducted and confirmed the effect of the reaction force on the sensitivity of the s-shape characteristic of the stress–strain curve. Finally, a polynomial regression was conducted and the calculated results based on the fourth-degree stress polynomial function correlated very well with the measured stress–strain curves.

## 1. Introduction

Fender structures are commonly installed at docks to prevent accidental damage to the vessel or dock side during berthing, so the fender material must have a high energy absorption capacity to minimize the reaction force. However, to extend the service life of the fender, the durability of the fender material is also an important concern. The fenders used in commercial docks, ports, and harbors are mostly fabricated from rubber because of its outstanding performances, like low transmission force and excellent energy absorption capacity. In addition, rubber has many more favorable properties such as high flexibility, good resilience, a long service life [1,2,3,4,5,6], an excellent resistance toward temperature [6,7,8,9,10,11,12], saltwater [13,14], and other environmental factors [15,16,17,18,19].

In practical applications, the reaction force and energy absorption capacity of the fender are very important. The energy absorption capacity is dominated by the geometry of the fender and the composition of the rubber from which it is made, and these two factors can be controlled during the design stage. This is because the geometry of the fender and the composition of the rubber lead to a change in the stress–strain response. Thus, it is possible to enhance the energy absorption capacity of the fender with an adequate geometric design and/or rubber material formula to maximize the designed transmission force that the fender can sustain. As well known, for buckling columns [20], the buckling strength of the straight column is expressed as $n\pi^{2}E{\left(K/L\right)}^{2}$, where *K* is the radius of gyration of the cross section ($K=\sqrt{I/A}$); *L* is the length of column; *n* represents the effective length, which depends on the boundary condition of the column; and *E* is the Young’s modulus. Moreover, area moment of inertia *I* should be about the axis of rotation of the cross section, and *A* is the cross-sectional area. Based on the two equations above-mentioned, it is clear that both the material and the profile of the buckling column directly affect the stress–strain response. Notably, the expression of the buckling strength is suitable and reasonable to explain the statement that the energy absorption capacity is dominated by the geometry of the fender and the composition of the rubber. Furthermore, it was found that the magnitude of the buckling strength decreased as the value of the radius of gyration of the cross section, *K*, was decreased. General rubber fenders have a hollow cross section to significantly improve the energy absorption capacity, and sometimes even leads to a slight increase in reaction. This is because the value of *K* of the rubber fender is decreased, so the buckling is induced easily, and thus the reaction force is maintained at more or less the same during compression.

For the hollow-cylinder rubber fenders, it is clear that they have a rotationally symmetric appearance, which guarantees a multi-directional energy absorption capability and a sturdy shear resistance. Therefore, the hollow-cylinder rubber fender has received some attention at the harbor in Taiwan. Moreover, under compressive loading, the hollow-cylinder fenders behave the same as a cylindrical buckling column (i.e., they absorb axial loads effectively and buckle radially). When the height and outer diameter of the hollow-cylinder rubber fenders are fixed and the specific composition of the rubber composites are required, then obviously the variation of the scale of the wall thickness yields a significant effect on the magnitude of the buckling strength and post buckling stress–strain response. Furthermore, it was asserted that the energy absorption capacity of the hollow-cylinder rubber fenders was critically dependent on the wall-thickness scale.

Therefore, this study conducted monotonic compressive tests to investigate the energy absorption capacity of hollow-cylinder rubber fenders with an original height ($H_{o})$ and outer diameter ($D_{o}$) of 100 mm and wall thicknesses ranging from 15–25 mm. The results confirmed that the extent of the lateral bending deformation in buckling depended on the wall-thickness scale. Furthermore, it was observed that for wall thicknesses of 20.0 mm or less, the measured reaction force caused the stress to have a rapid growth at small deformations, was then maintained at high level, and slightly increased/decreased over a large strain-range in the post-buckling condition, before significantly increasing once again as the strain further increased. In other words, a clear s-shape response was observed in the stress–strain curve. For larger wall thicknesses, the measured stress also increased with an increasing compressive strain, but the s-shape was vague. Overall, the results showed that the extent of the s-shape characteristic in the stress–strain curve was highly sensitive to the wall-thickness scale over the considered range of 15–25 mm.

This study asserted that the sensitivity of the s-shape response could be attributed to the applied compressive deflection inducing the folded inner surfaces of the cylindrical fender to self-contact, where the size of the folded contact area depended on the applied compressive deflection and the scale of wall-thickness. Based on this assertion, it was further speculated that the self-contact area produced an additional reaction force, whose magnitude also depended on the extent of the lateral bending deformation. In other words, the measured load was equal to the resultant of the reaction force in the contact area and the one from the occurrence of compression-bending deformation in the side wall of the hollow fender. The inference revealed that the stress–strain response of the cylindrical fender reflected not only the reaction force produced in the thickness of the cylinder wall under the effects of the applied compressive load, but also an additional reaction force at the self-contact area whose magnitude varied with the scale of the applied compression deflection. The validity of this assertion was investigated by a finite element analysis (FEA) in which the hyperelastic Mooney–Rivlin material model was given to the hollow fender. It was shown that the simulation results for the global buckling of the fender and the local buckling of the cylinder ends were consistent with the experimental observations and the measured stress–strain curves. Moreover, the results confirmed the effect of the additional reaction force produced by the self-contact area on determining the extent of the s-shape characteristic in the stress–strain curves. To fit the measured $E_{s}-e_{c}$ curves obtained from the monotonic compression tests, this study used a polynomial regression analysis technique to model the relationship between the strain-energy density,$\;E_{s}$, and the compressive strain, $e_{c}$, of the hollow-cylinder fenders with different wall thicknesses. Through a process of trial-and-error, it was found that the measured $E_{s}-e_{c}$ curves could be accurately modeled by using a fifth-degree energy polynomial function. Furthermore, the validity of the corresponding fourth-degree stress polynomial function for the measured stress–strain curve was also confirmed.

## 2. Experiment

**Figure 1:**
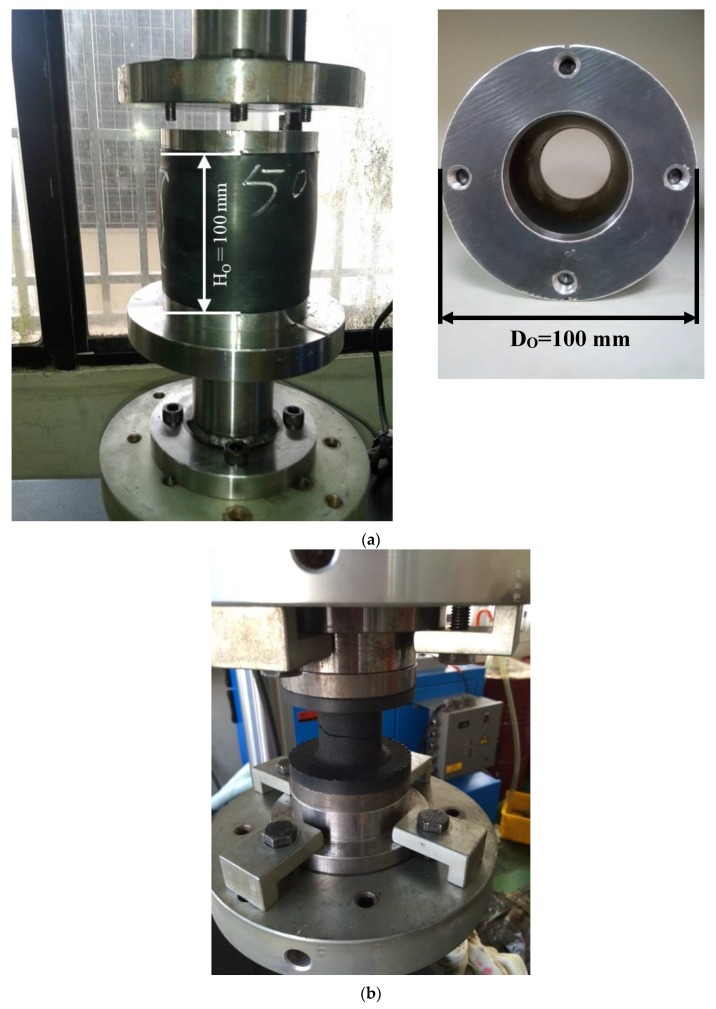

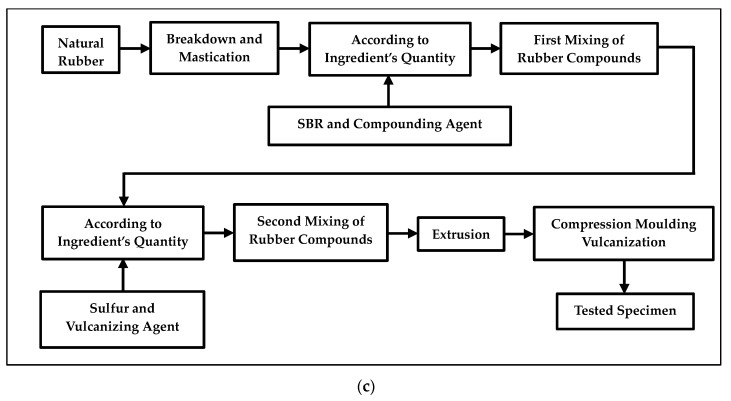
(**a**) Geometry and dimensions of tested hollow-cylinder rubber fenders, and (**b**) experimental setup for monotonic tension tests, and (**c**) the manufacturing processes of the rubber fender provided by the supplier.

Figure 1a shows an un-deformed scaled hollow-cylinder rubber fender with a height, $H_{o}$, and diameter, $D_{o}$, of 100 mm. Monotonic compression tests were performed to a final strain of 50% to examine the effect of the wall-thickness scale, $T_{k}$, on the compressive response of the fender.

In this study, the hollow-cylinder fenders with wall thicknesses of 25.0, 22.5, 20.0, 17.5, and 15.0 mm were used to perform the tests. In addition to the monotonic compression tests, the fully compressive cyclic tests ($A_{\Delta }=-1$) with a strain range of $\Delta e_{c}=48\%$ were also conducted to observe the wall-thickness effect on the durability of the fender. Monotonic tension tests were additionally performed on the solid cylindrical rubber sample shown in Figure 1b with a height of 35 mm and a diameter of 40 mm to determine the material coefficients required for the Mooney–Rivlin constitutive model.

All of the samples (both compression and tensile) were purchased from Chin-Cheng Rubber Factory, Chiayi, Taiwan. The manufacturing processes of the hollow-cylinder rubber fender are shown in the flowchart in Figure 1c. According to the manufacturer’s specifications, the Shore A hardness of the samples was 66. The composition of the fender samples is summarized in Table 1, in which the natural rubber and styrene butadiene rubber (SBR) are the main ingredients of the fender, and the others are the additives used to strengthen the mechanical properties and enhance the environmental resistance of the rubber fender.

Monotonic compression tests were performed at a constant speed of 0.1 mm/s until the measured deformation, $\delta_{c},$ reached a value of −50 mm. On the other hand, the tensile tests were conducted using a constant crosshead rate of 0.01 mm/s and were continued until the extension reached 21 mm. In the cyclic straining tests, the sinusoidal waveform with a frequency of 0.31 Hz was used. All tests were performed in the stroke-controlled mode. In addition, Max software (Version 7.0, Instron, Norwood, MA, USA), integrated with the testing system, was used to record the measured load-deformation points throughout the compression and tension tests to construct the corresponding stress–strain plots.

## 3. Test Results and Observations

### 3.1. Observations of Compressive Deformation

**Figure 2:**
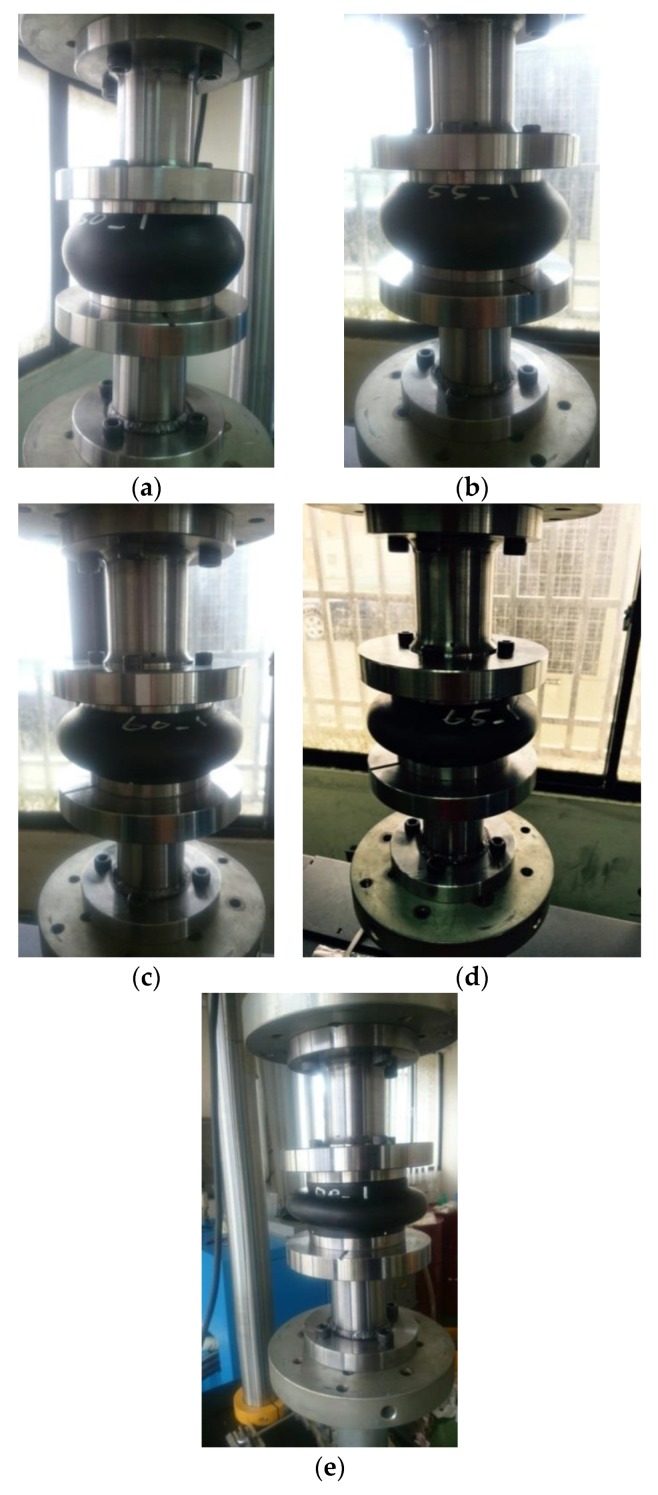
Experimental observations of the tested hollow-cylinder rubber fenders with different wall thicknesses under maximum compressive deformation (50 mm): (**a**) 25.0, (**b**) 22.5, (**c**) 20.0, (**d**) 17.5, and (**e**) 15.0 mm.

Figure 2a–e shows the deformations of the hollow-cylinder fenders with wall thicknesses of 25.0, 22.5, 20.0, 17.5, and 15.0 mm, respectively, during monotonic compression tests. All of the fenders buckled under the applied compressive strain. However, a progressive change in the lateral bending deformation was observed as the wall thickness increased. For fenders with larger wall thicknesses of 25.0 and 22.5 mm, respectively, both the global buckling of the fender and the local buckling at the fender ends were relatively small. However, for fenders with wall thicknesses of 20.0, 17.5, and 15.0 mm, both buckling effects were more pronounced. Generally, the scale of the lateral bending deformation increased only slowly at the beginning of compression, but then increased quickly until the applied compressive deflection reached a value of approximately $e_{c}=30\%$, before increasing once again for the performed monotonic compression tests in this study.

**Figure 3:**
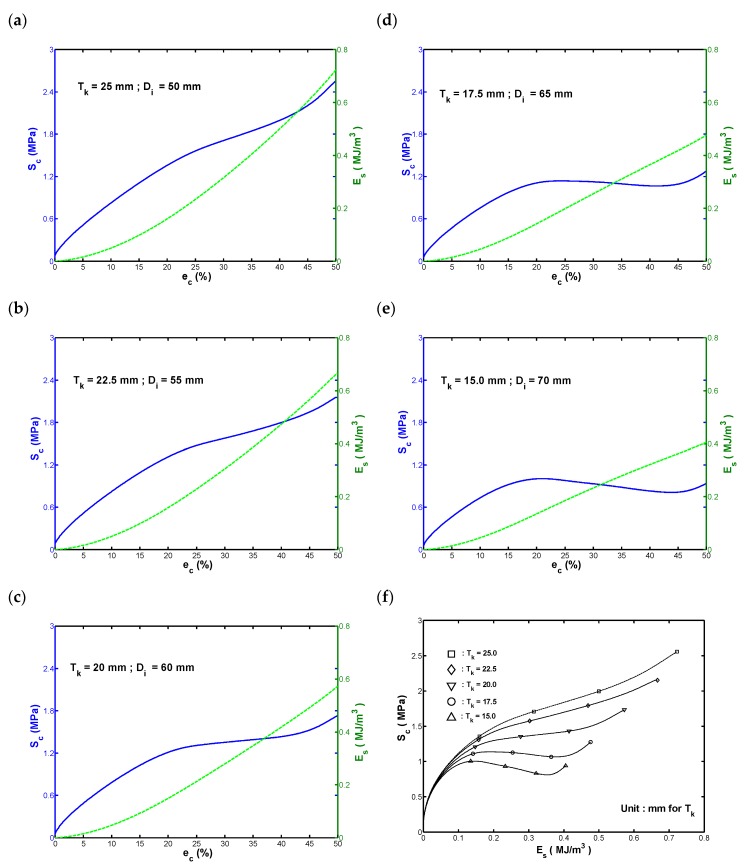
Monotonic compressive responses of hollow-cylinder rubber fenders with wall thicknesses of: (**a**) 25.0, (**b**) 22.5, (**c**) 20.0, (**d**) 17.5, and (**e**) 15.0 mm. (**f**) Comparison of $S_{c}-E_{s}$ curves for fenders with different wall-thicknesses.

To obtain further insights into the compressive deformation behavior of the cylindrical fenders with different wall thicknesses, the measured transmission stress, $S_{c}$, was plotted against the compressive strain,$\;e_{c}$, for each fender, as shown in Figure 3a–e. In constructing the stress–strain curves, the transmission stress and compressive strain were computed respectively as:

(1) $$S_{c}=\frac{\left|P_{c}\right|}{A_{o}}$$

and

(2) $$e_{c}=\;\frac{\left|\delta_{c}\right|}{H_{o}}$$

where $P_{c}$ and $\delta_{c}$ are the measured load and compressive displacement, respectively. Note that the absolute forms of the $S_{c}$ and $e_{c}$ were used for an easier comparison with the experimental observations. In accordance with first principles, the absorbed strain energy per unit volume of the rubber fender is given by the area under the $S_{c}-e_{c}$ curve, which can be calculated by Equation (3), where $E_{s}$ is the strain-energy density.

(3) $$E_{s}=\int_{0}^{e_{c}}S_{c}de_{c}$$

For each of the measured $S_{c}-e_{c}$ curves, the corresponding value of $E_{s}$ was calculated and added to Figure 3a–e. In practice, the energy absorption capacity and corresponding transmission force are key parameters in the design of hollow-cylinder rubber fenders. Hence, the $S_{c}-e_{c}$ curves and $E_{s}-e_{c}$ curves as shown in Figure 3a–e effectively represent the performance curves of the rubber fenders in this study. As shown in Figure 3a–e, the stress–strain curves of the deformed cylindrical fenders exhibited an s-shape profile. Moreover, the extent of this s-shape characteristic became clearer as the wall thickness was reduced from 25 to 15 mm. The s-shape response in the stress–strain curves indicates that a gradual change in bending from a downward direction to an upward direction as the compression proceeds. In other words, the increment of the measured stress ($\Delta S_{c}$) changes from negative to positive as the compressive strain increases in the range from 0 to 50.

Observing the experimental results shown in Figure 2a,b and the corresponding stress–strain curves shown in Figure 3a,b, no obvious improvement in the energy absorption performance was detected as the wall-thickness scale was reduced from 25.0 to 22.5 mm, because both cases led to similar global buckling of the fender and local buckling at the cylinder ends. However, it was found that the axial stiffness of the fender with wall thicknesses of 22.5 mm decreased about 16% in comparison with the one with a wall thickness of 25.0 mm. The stress–strain curves shown in Figure 3c–e correspond to wall thicknesses of 20, 17.5, and 15.0 mm, respectively, and these three cases exhibited a clearer s-shape characteristic than those with greater wall thicknesses. In the three cases, the measured transmission stress increased rapidly from zero, but then remained approximately constant or fell slightly to a local minimum as the strain further increased, and then rose again. The plateau region in the s-shape curve implies that the fender has a high level of admissible stress (i.e., following the buckling event, the fender still can absorb a high transmission stress). In other words, three fenders exhibit a high energy absorption capacity, and hence a low transmission force. In general, local maximum stress in the s-shape curves shown in Figure 3d,e represent the buckling strengths of the corresponding fenders. From inspection, the buckling strength of the fender with a wall thickness of 17.5 mm was found to be 1.1388 MPa, while that of the fender with a wall thickness of 15.0 mm was 1.007 MPa. Based on the above observations in Figure 2c–e and Figure 3c–e, it is evident that the three fenders exhibited a high energy absorption capacity, and hence a low transmission force.

For cylindrical fenders investigated in this study, the energy absorption capacity of the fender under a certain specified transmission force is very important to the maritime industry to protect both the dock and the ship from accidental damage during berthing. Therefore, the transmission stress, $S_{c}$, against the strain-energy density, $E_{s}$, for each of the five cylindrical fenders was plotted and is shown in Figure 3f. It can be seen that the $S_{c}-E_{s}$ curve shifted upward and the range of $E_{s}$ increased when the wall-thickness scale increased. In other words, for a specified $E_{s}$, a higher level of transmission stress is induced by the fenders with a greater wall thickness.

**Figure 4:**
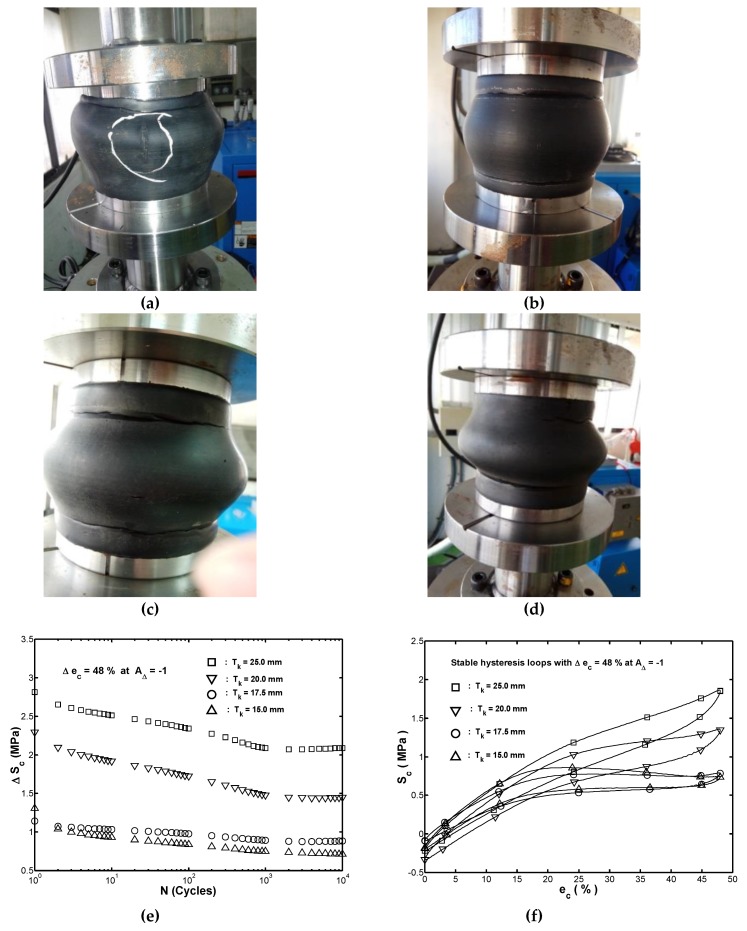
Deformations of the tested sample after cyclic compression tests: (**a**) $T_{k}=25.0\;\mathrm{mm}$ at the cyclic loading number of 11,894; (**b**) $T_{k}=20.0\;\mathrm{mm}$, (**c**)$\;T_{k}=17.5\;\mathrm{mm}$, and (**d**)$\;T_{k}=15.0\;\mathrm{mm}$ at the cyclic loading number of 10,000; (**e**) Relationship between the stress range and number of cycles; (**f**) Stable hysteresis loops in cyclic compression tests with a strain range of $\Delta e_{c}=48\%$ for four different wall-thickness scales.

Figure 4a–d show the deformations of the tested samples with wall thicknesses of 25.0, 20.0, 17.5, and 15.0 mm, respectively, where the tested samples experienced 11,000 loading cycles except for the one shown in Figure 4a, which was obtained at the loading cycle number of 11,894.

In Figure 4a, it was found that a clear axial fatigue crack occurred on the surface of the tested sample. Moreover, for the tested samples shown in Figure 4b–d, no cracks were detected. The observation confirms that the wall-thickness scale had a significant effect on the durability of the hollow-cylinder rubber fender subjected to a fully compressive cyclic straining with a strain range of $\Delta e_{c}=48\%$. Figure 4e shows a plot of the stress range, $\Delta S_{c}$, with the applied cycles, N, for the tested fender with four different wall-thicknesses considered in the cyclic compression tests. It was found that the magnitude of $\Delta S_{c}$ decreased progressively as the number of cycles increased until it reached 1000 cycles and then remained approximately constant thereafter. The observation confirmed that the extent of decrease in $\Delta S_{c}$ depends on the wall-thickness scale. Based on the results shown in Figure 4e, the stress–strain hysteresis loops obtained at the ten-thousandth cycle are thus used to represent the stable behavior of the rubber fender. Figure 4f shows the stable stress–strain hysteresis loops with four different wall-thicknesses obtained from the recorded data corresponding to the ten-thousandth cycle of the cyclic compression tests. As shown in Figure 4f, it was found that when the wall-thickness scale increased, the closed hysteresis loop shifted to higher stress and the closed area within the hysteresis loop also increased. This finding indicates that the energy absorption capacity of the hollow-cylinder fender increases with an increasing wall-thickness.

### 3.2. Estimation on Efficiency of Rubber Fender

**Figure 5:**
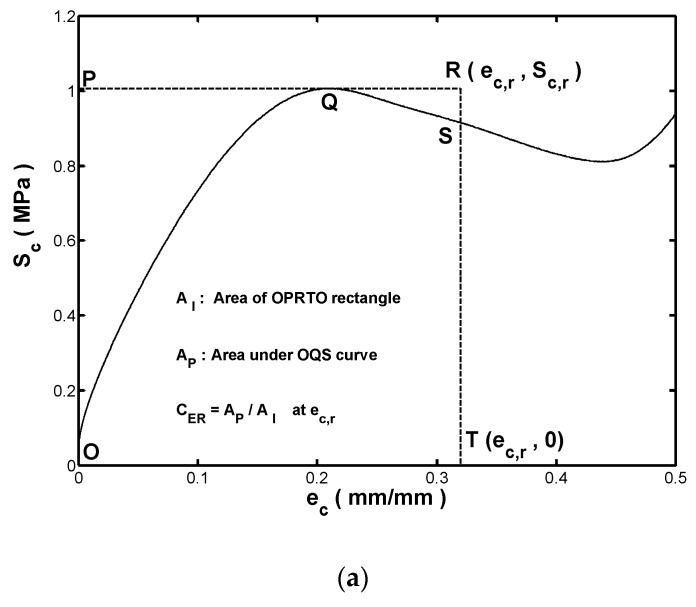

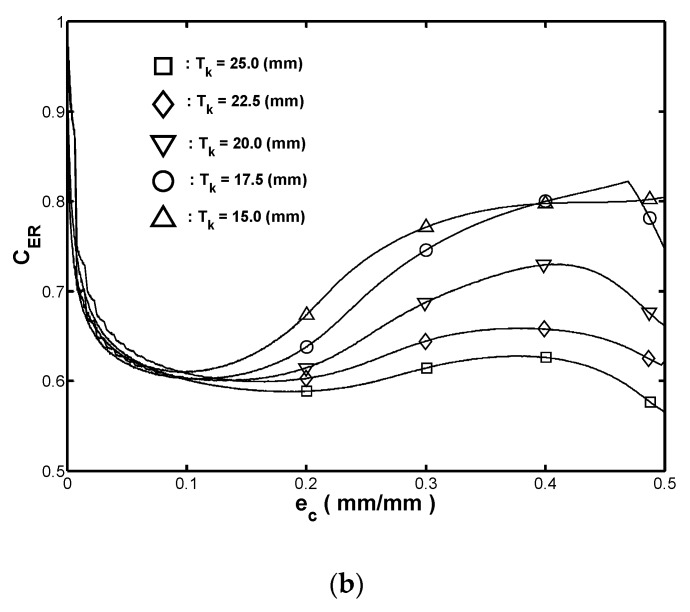
(**a**) Illustration of proposed energy-effectiveness index $C_{ER}$; (**b**) Variation of $C_{ER}$ with compressive strain $e_{c}$ for fenders with different wall thicknesses.

Figure 5a shows the stress–strain response of an ideal rubber fender during compressive straining, in which $e_{c,r}$ and $S_{c,r}$ are shown in the upper-right corner of the figure, where $S_{c,r}$ represents the stress of the ideal rubber fender as the strain increases from 0 to $e_{c,r}$. In accordance with first principles, As shown in Figure 5a, the absorbed strain-energy density of the ideal fender is equal to the area enclosed by the OPRTO rectangle denoted as $A_{I}$. For a practical (i.e., non-ideal) fender, the stress–strain response is indicated by the OQS curve, and the actual absorbed strain-energy density ($A_{P}$) is given by the area under the OQS curve. In addition, the maximum stress induced in the practical fender is equal to the ideal value $S_{c,r}$ located at point Q. By definition, the ideal fender achieves a better energy absorption capacity over the strain range from 0 to $e_{c,r}$ than the practical fender. To facilitate the discussions, this study introduced an energy-effectiveness index, $C_{ER}$, to quantify the energy absorption efficiency of the investigated fenders with different wall thicknesses. Referring to Figure 5a, the energy-effectiveness index $C_{ER}$ is defined as:

(4) $$C_{ER}=A_{P}/A_{I}\;\mathrm{at}\;a\;\mathrm{specified}\;\mathrm{value}\;\mathrm{of}\;e_{c,r}$$

where a higher $C_{ER}$ indicates that the stress–strain response of the practical fender is closer to that of the ideal fender, meaning that a fender with higher $C_{ER}$ index provides a greater energy absorption capacity under a given transmission force.

$C_{ER}$ was plotted against the compressive strain $e_{c,r}$ for the five fenders and the results are shown in Figure 5b. For all of the fenders, $C_{ER}$ reduced rapidly with an increasing compressive strain until $e_{c,r}=0.1$. Afterward, $C_{ER}$ gradually increased to a local maximum value as the compressive strain rose. For fenders with wall thicknesses of 25.0, 22.5, and 20.0 mm, respectively, the maximum value of $C_{ER}$ occurred at a compressive strain of about 0.4. The value of $C_{ER}$ then reduced noticeably at the final recorded strain of $e_{c}=0.5$. It was noted that the rise in the $C_{ER}$ as $e_{c,r}$ increased from 0.1 to 0.4 was more pronounced in the fenders with a smaller wall thickness. From the $C_{ER}-e_{c}$ curve of the fender with a wall thickness of 17.5 mm, it can be seen that $C_{ER}$ increased to a sharp peak of about 0.8 at a strain of approximately 0.47 before reducing to around 0.75 as the compressive strain further increased to 0.5. Therefore, it was inferred that the maximum stress at the final recorded strain was not equal to the buckling strength. It was further noted that among all of the fenders, the one with the smallest wall thickness (15 mm) had the highest $C_{ER}$ at the final recorded strain. In other words, the buckling strength of the fender represents the maximum stress response over the applied strain range.

Overall, the results presented in Figure 5b show that the $C_{ER}$ increased with a decreasing wall thickness during compression straining. In other words, for the hollow-cylinder fenders considered in this study with wall-thickness scales ranging from 15 to 25 mm, the energy absorption capacity increased with a decreasing wall thickness. In general, the results indicate that the proposed energy-effectiveness index $C_{ER}$ provides a convenient approach for comparing the energy absorption efficiencies of different fenders for a given design requirement of transmission force.

### 3.3. Estimation on Compression Properties of Rubber Fender

As shown in Figure 3f, for fenders with a wall thickness of 17.5 mm or more, the maximum values of $E_{s}$ and $S_{c}$ occurred at the final recorded strain of $e_{c}=50\%$. However, for the fender with the smallest wall thickness of 15.0 mm, the stress at the maximum compressive strain of 50% was slightly lower than the buckling strength. From the design viewpoint, the magnitude of the transmission stress under the maximum desired compression is important. For convenience, this study denoted the value of $S_{c}$ at the maximum compressive strain $(e_{c}=50\%$) as the compression strength, $S_{c,c}$. Furthermore, the strain-energy density at the corresponding strain was denoted as the compression toughness, $U_{c,\;c}$. The measured mean values of compression strength/toughness are summarized in Table 2, where a diameter ratio $R_{D}$ is used to represent the ratio of the inner diameter of the hollow-cylinder fender $D_{i}$ to the outer diameter $D_{o}$, and a larger value of $R_{D}$ indicates a smaller wall thickness.

**Figure 6:**
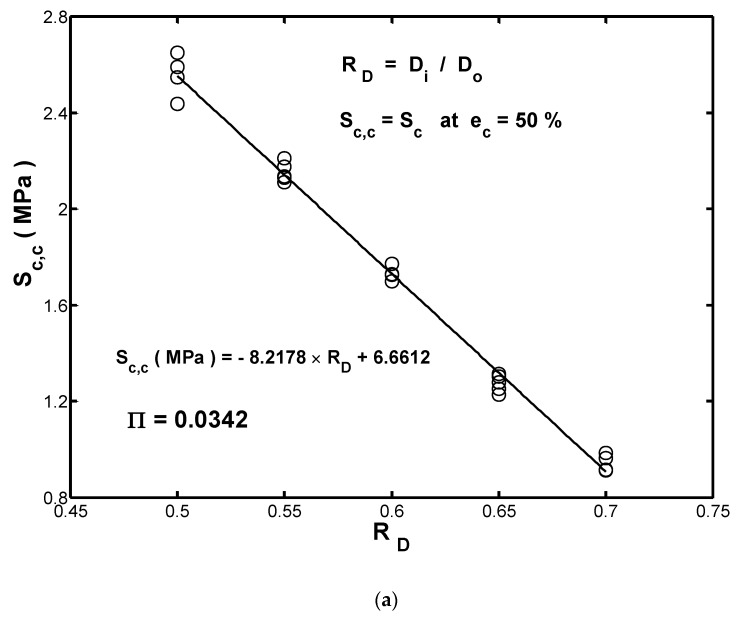

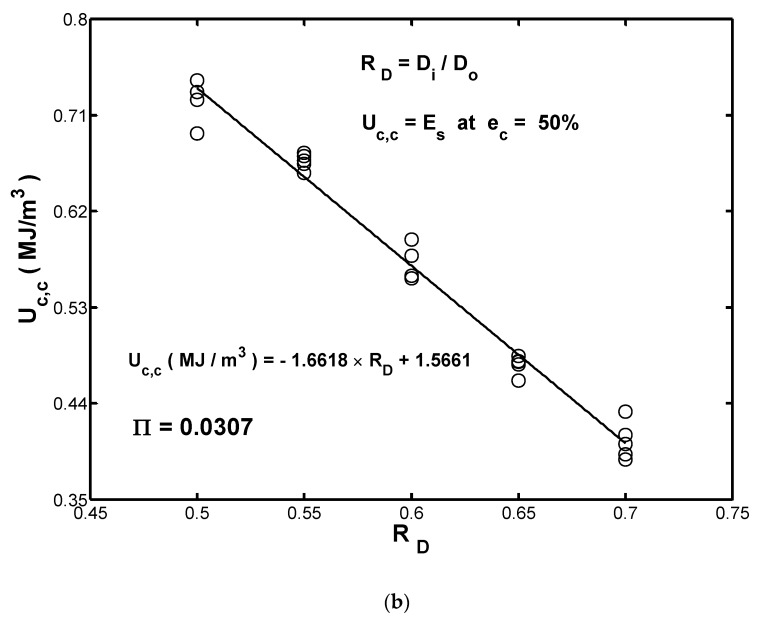
Measured data and linearly fitted results for (**a**) =compression strength $S_{c,c}$ and (**b**) compression toughness $U_{c,c}$ for fenders with a diameter ratio $R_{D}\;$ ranging from 0.5 to 0.7.

The measured values of $S_{c,c}$ and $U_{c,\;c}$ were plotted against $R_{D}$ and are shown in Figure 5a,b, respectively. An ordinary least-squares (OLS) regression analysis to the measured strength values was conducted and the result is added in Figure 6a, in which it was found that $S_{c,c}$ can be modeled by the following function of $R_{D}$:

(5) $$S_{c,c}\left(\mathrm{MPa}\right)=\;-8.2178\times R_{D}+6.6612$$

Similarly, the compression toughness $U_{c,c}$ can be modeled as

(6) $$U_{c,c}\left({\mathrm{MJ}/m}^{3}\right)=\;-1.6618\times R_{D}+1.5661$$

As shown in Figure 5a,b, both fitted curves showed good agreement with the measured results. Therefore, Equations (5) and (6) provide a convenient method of estimating the compression strength and toughness of the hollow fenders for wall thicknesses in the range of 15–25 mm without the need for further experiments. Figure 5a,b show that the compression properties of the fenders decreased with an increasing value of $R_{D}$ (i.e., a decreasing wall thickness). In other words, the result confirms that the wall-thickness has a significant effect on both the compression strength and the compression toughness of the hollow-cylinder rubber fenders investigated in this study.

## 4. Discussion

### 4.1. Determination of Material Coefficients for Mooney-Rivlin Model

As described in Section 3.1, the extent of the s-shape characteristic in the stress–strain curves depends significantly on the scale of the wall thickness. In this study, it was asserted that the effect of the wall thickness on the profile of the s-shape curve could be attributed to the self-contact of the buckled cylindrical fenders. More specifically, in the post-buckling condition, the inner surface of the fenders collapsed and folded on itself to form a self-contact area. This contact area increased with an increasing compressive strain. The formation of this self-contact area generated an additional reaction force and resulted in a progressive change in the measured $\Delta S_{c}$ when the compressive strain increased. Since the contact area occurred on the inner surface of the hollow cylindrical fender, the assertion above could not be confirmed experimentally. Therefore, the folding effect and the increase in the reaction force were investigated by a finite element analysis (FEA). Previous studies [2,21,22,23,24,25,26,27,28,29] have shown that the hyperelastic Mooney–Rivlin material model provides an accurate assessment of the constitutive behavior of many products or structures made of rubber-like materials under various loadings, so the Mooney–Rivlin material model was also used in the FEA in this study.

The Mooney–Rivlin model is derived from the stress–strain relationship for hyperelastic materials with a known strain state. The model further assumes that the local strain-energy density for an incompressible solid can be expressed as a simple function of the local strain invariants. In this study, the material coefficients for the Mooney–Rivlin model used in the hollow-cylinder fenders were determined by a standard tensile test on a rotationally-symmetric specimen, as shown in Figure 1b. For uniaxial extension, the relationship between the applied force F and the resulting extension ΔH for a true Mooney–Rivlin material model is given by:

(7) $$F/A_{o}\;=\;2\left(C_{10}+C_{01}/\lambda \right)\left(\lambda -1/\lambda^{2}\right)$$

With

(8) $$\lambda =\left(H_{o}+\Delta H\right)/H_{o}$$

where $A_{O}$ is the original cross-sectional area of the specimen ($A_{o}=\left(\pi /4\right){\left(35\right)}^{2}\;({\mathrm{mm}}^{2}$))$;\;H_{0}$ is the reference length ($H_{o}=40\mathrm{mm}$); and λ is the relative elongation. Furthermore, $C_{10}$ and $C_{01}$ are the two material coefficients needed to input in the FEA, and are determined by fitting Equations (7) and (8) to the experimental results. In general, the test data obtained from tensile tests are delivered in a form that is independent of the geometry of the specimen used. Many other possible formats are also available [2,30,31]. This study adopted the engineering stress $S_{i}$, which represents the force per unit reference area such as

(9) $$S=F/A_{o}$$

For the Mooney–Rivlin material model, the engineering stress at a given value of the relative elongation can be modeled as:

(10) $$S\left(\lambda \right)=2\left(C_{10}+C_{01}/\lambda \right)\left(\lambda -1/\lambda^{2}\right)$$

Given *N* pairs of measurements, $\left(\lambda_{i},S_{i}\right)\left(i=1\ldots N\right)$, the values of $C_{10}$ and $C_{01}$, which provide the best fit to the measured data, are those that minimize the total squared error as follows:

(11) $$e=\;\overset{N}{\underset{i=1}{\sum }}{\left(S\left(\lambda_{i}\right)-S_{i}\right)}^{2}$$

For the rubber sample shown in Figure 1b, the material coefficients were determined to have the following values:

(12) $$C_{10}=0.176\;\mathrm{MPa}\;\mathrm{and}\;C_{01}=0.686\;\mathrm{MPa}$$

**Figure 7:**
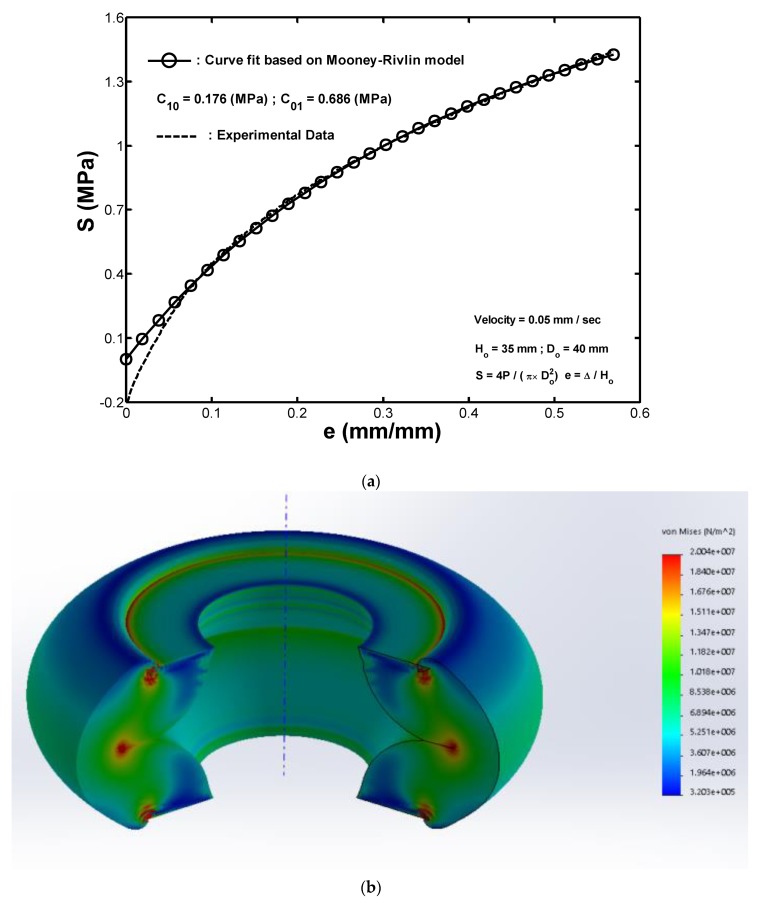

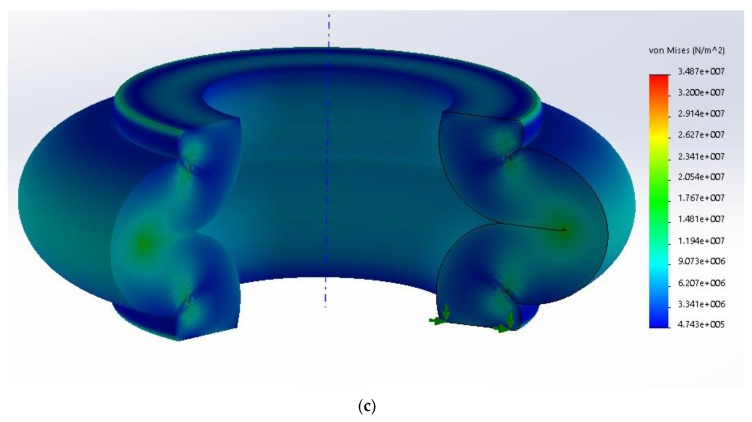
(**a**) Comparison of the experimental stress–strain curve of the rubber fender under tensile loading and the fitted one obtained from the Mooney–Rivlin model. Finite element analysis results for the stress distribution contour of the rubber fender with wall thicknesses values of (**b**) 25.0 and (**c**) 15.0 mm under a maximum compression displacement of 50 mm.

Figure 7a compares the experimental results for the stress–strain response of the rubber sample with the fitted results obtained by using the Mooney–Rivlin model. It can be seen that despite the small deviation that occurred at the initial loading stage, the predicted stress–strain values of the rubber sample were in close agreement with the experimental values. Therefore, the validity of the Mooney–Rivlin model as the basis for the FEA was confirmed.

### 4.2. Simulation Results Based on Mooney-Rivlin Material Model

An FE model of a hollow-cylinder rubber fender was created in SolidWorks with the dimension of a height of 100 mm, an outer dimeter of 100 mm, and a wall thickness of 25 mm. The hollow-cylinder rubber fender is an axially symmetric structure. Therefore, to save on simulation time, the finite element analysis was conducted on a planar rectangle, which represents the axial cross section of the fender. After the analysis, the function axial-symmetric mirror function in the post-processing module was used to revolve the rectangle so that the deformation of the whole hollow-cylinder rubber fender could be presented, as shown in Figure 5b.

The FE model of the planar rectangle is meshed by 1^st^-order 2D shell elements with an average mesh size of 1.5 mm. The whole FE model consisted of 2058 elements and 4281 nodes. The material given to the model was the hyperelastic Mooney–Rivlin model, in which the two material coefficients $C_{10}=0.176\;\mathrm{MPa}$ and $C_{01}=0.686\;\mathrm{MPa}$ are given. To calculate the self-contact of the planar rectangle, the surface-to-surface contact was used and frictionless was chosen. Furthermore, in the pre-processing of FEA, the lower end of the cylinder (the bottom of the planar rectangle) was fixed and the upper end (the top of the planar rectangle) was given an enforced displacement of 60 mm vertical downward to compress the cylinder. The simulation was terminated at the applied compressive displacement of 51.6 mm due to the structural instability induced by the local buckling at both cylinder ends. The other reason for termination was that the self-contact condition at both ends also failed.

For fender models with wall thicknesses of 25 and 15.0 mm, Figure 6b,c respectively show the simulation results of the stress distribution contour within the cylinder under the compressive displacement of 51.6 mm. The results confirm that the fender buckled under the effects of the applied strain and the inner surface experienced a folding-induced self-contact effect. Notably, the regions of local buckling, as shown in Figure 7b, at the two ends of the cylinder did not self-contact, whereas the self-contact phenomenon was obvious in the regions of local buckling, as shown in Figure 7c. Hence, the regions of global/local buckling and the occurrence of the self-contact at both ends of the fender depend on the scale of the wall thickness. The simulation results for the buckling behavior of the cylinder are consistent with the experimental ones shown in Figure 2a,e.

The FEA was repeated for fender models with wall thicknesses of 22.5, 20.0, and 17.5 mm, respectively. The fender models with wall thicknesses of 22.5 and 20.0 mm also show self-contact under the effects of global buckling, and the global buckling region increased with a decrease in the scale of wall thickness. Similarly, the local buckling region at both ends of the fender also showed the self-contact behavior, which was slightly increased with the decrease in the scale of wall thickness. These observations explain the absence of a local maximum load (buckling strength) in the stress–strain curves shown in Figure 3b,c, because the self-contact area strengthens the global axial stiffness of the fender. As a result, the local buckling effect has a slight contribution to the stress–strain response. In general, the simulation results confirm that the variation of the slope of the stress–strain curves from negative to positive in Figure 3a–c can be attributed to the effects of an additional reaction force produced by the self-contact area almost induced under the global buckling.

Similarly, the FEA results of the fenders with smaller wall thicknesses of 17.5 and 15.0 mm showed that the region of global/local buckling increased significantly in both cases, and resulted in a delay in the development of the folded area on the inner surface. In other words, the occurrence of a considerable buckling region led to a clearer s-shape characteristic in the stress–strain curve. More specifically, the local buckling region shifted toward the center (i.e., mid-height position) of the compressed fender and resulted in a more intense self-folding effect (i.e., a greater self-contact area). Overall, the simulation results confirmed the assertion of this study that the sensitivity of the stress–strain response of the hollow-cylinder fenders to the wall thickness can be attributed to the additional reaction force produced by the self-contact area of the inner surface, where the magnitude of this reaction force depends on the scale of the wall thickness.

### 4.3. Modeling of Stress–Strain Response by Using Energy Polynomial Function 

Polynomial functions provide a simple and efficient approach for fitting the monotonic curves describing the nonlinear relationship between an independent variable and multiple dependent variables [32]. However, to minimize the computational effort when one uses the polynomial function for prediction, it is necessary to minimize the degree of the polynomial function while still preserving an acceptable consistency between the fitted curve and the actual one.

An observation of the measured $\left(e_{c},\;E_{s}\right)$ data in Figure 3a–e confirm that $E_{s}$ increases monotonically with an increasing strain over the range of $e_{c}=0–50\%$. Therefore, this study applied a polynomial regression approach to model the relationship between $E_{s}$ and $e_{c}$ as an *n*-th degree polynomial function for each of the measured $E_{s}-e_{c}$ curves shown in Figure 3a–e. Based on the results obtained from trial-and-error least-square fitting tests, it was found that a fifth-degree polynomial (i.e., *n* = 5) was sufficient to provide a good fit to the measured $\left(e_{c},\;E_{s}\right)$ data in every case. The corresponding polynomial regression coefficients are listed in Table 3. For convenience, the polynomial function $E_{s}\left(e_{c}\right)$ is called the energy polynomial function hereafter and is given by Equation (13).

(13) $$E_{s}\left(\mathrm{MJ}/m^{3}\right)=C_{5}\times e_{c}{}^{5}+C_{4}\times e_{c}{}^{4}+C_{3}\times e_{c}{}^{3}+C_{2}\times e_{c}{}^{2}+C_{1}\times e_{c}+C_{o}\;;e_{c}\;\left(\mathrm{mm}/\mathrm{mm}\right)$$

From a physical viewpoint, the strain-energy density $E_{s}$ and the transmission stress $S_{c}$ are related as

(14) $$S_{c}=dE_{S}/de_{c}$$

According to Equation (14), the value of $S_{c}$ at any given value of $e_{c}$ in the range of 0–50% can be estimated directly if the energy polynomial function is available. In other words, a further fourth-degree polynomial function can be derived for $S_{c}$ in terms of variable $e_{c}$. For convenience, the resulting polynomial function, $S_{c}\left(e_{c}\right)$, is denoted as the stress polynomial function in the remainder of this study.

**Figure 8:**
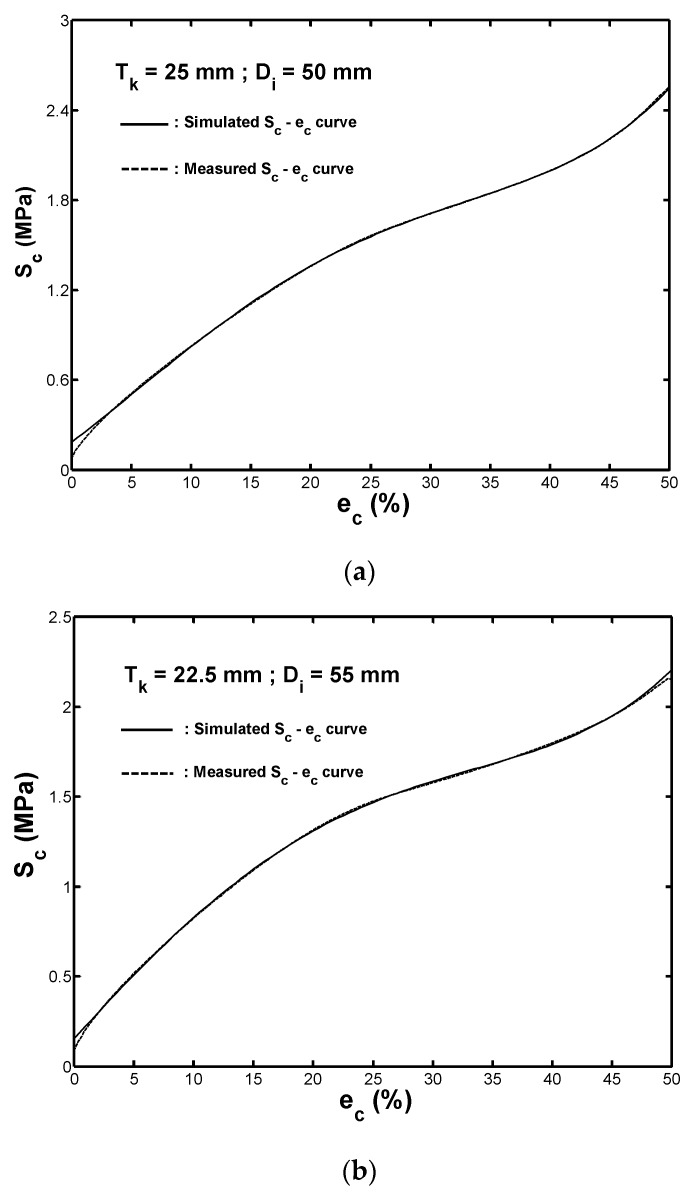

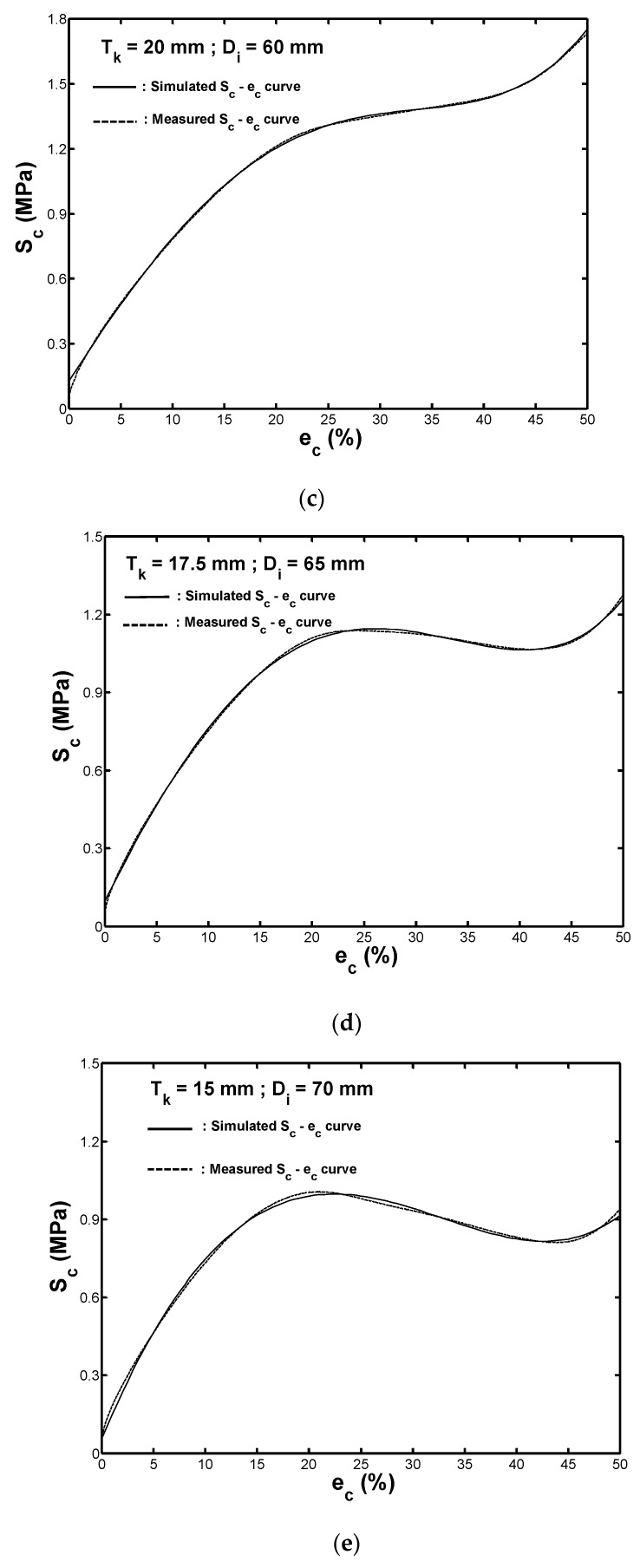
Comparison of the measured $S_{c}-e_{c}$ curves and simulated ones under monotonic compression loading for fenders with wall thicknesses of: (**a**) 25.0 mm, (**b**) 22.5 mm, (**c**) 20.0 mm, (**d**) 17.5, and (**e**) 15.0 mm.

Figure 8a–e compare the measured $S_{c}-e_{c}$ curves for the five rubber fenders with different wall thicknesses with the simulated ones obtained by using the proposed stress polynomial function based on Equation (14) and the coefficient values are summarized in Table 3. A good fit was observed between the simulated curves and the measured ones in all cases. Therefore, the stress polynomial function provides an accurate assessment of the stress–strain response of the hollow fenders with wall thicknesses ranging from 15 to 25 mm.

**Figure 9:**
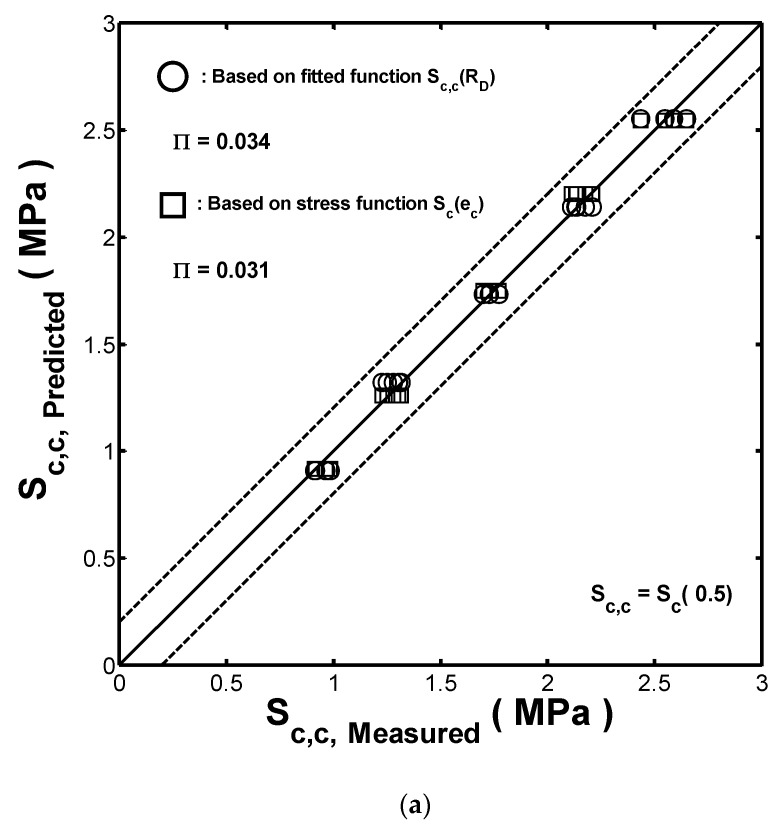

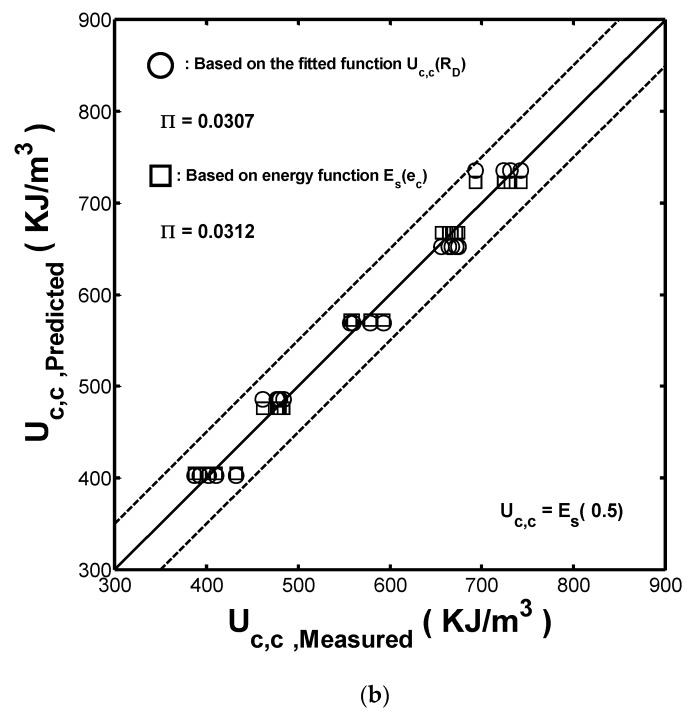
Comparison of the measured and predicted values of (**a**) strength and (**b**) toughness of the fenders with different wall thicknesses. Note that the predicted values of the compression strength and compression toughness are obtained from both the stress/energy polynomial functions and the fitted functions, $S_{c,c}\left(R_{D}\right)$/$U_{c,c}\left(R_{D}\right)$, for comparison purposes.

Figure 9a compares the measured compression strengths ($S_{c,c}$) of the five fenders with the estimated ones obtained by using the linear fitted function $S_{c,c}\left(R_{D}\right)$ and the stress polynomial function $S_{c}\left(e_{c}\right)$, respectively. Similarly, Figure 9b compares the measured compression toughness$\;U_{c,c}$ with the estimated ones obtained by the linearly fitted function $U_{c,c}\left(R_{D}\right)$ and the energy polynomial function $E_{s}\left(e_{c}\right)$, respectively. From a detailed inspection, the error bands in Figure 8a,b have ranges of ±0.25 (MPa) and $\pm 50\;\left(\mathrm{KJ}/m^{3}\right)$, respectively. Hence, both the proposed stress/energy polynomial functions and the linearly fitted functions $S_{c,c}\left(R_{D}\right)$/$\;U_{c,c}\left(R_{D}\right)$ provide satisfactory estimations of the compression properties (strength and toughness) of the hollow-cylinder rubber fenders. To compare the calculated strengths of the linear fitting and those of the polynomial function methods, respectively, the following evaluation metric was introduced:

(15) $$\Pi =\sqrt{\frac{\sum \left(\Delta_{i}-\Delta_{i,\mathrm{mean}}\right)}{n-1}}$$

where $\Delta_{i}$ is the ratio of the measured value to the predicted value, and $\Delta_{i,\mathrm{mean}}$ is the average value of all the calculated values of $\Delta_{i}$ (note that n=24 in both cases).

Applying Equation (15) to the prediction results given in Figure 9a, it can be found that the stress polynomial function (with Π=0.031) provides a better prediction of the compression strength of the present hollow-cylinder fenders than the fitted function (with Π=0.034). In contrast, in Figure 9b, the fitted function $U_{c,c}\left(R_{D}\right)$ provides a more accurate estimation of the compression toughness of the considered fenders than the energy polynomial function (i.e., Π=0.0307 and Π=0.0312, respectively).

## 5. Conclusions

This study investigated the stress–strain response and the buckling behavior of hollow-cylinder rubber fenders with wall thicknesses in the range of 15–25 mm by conducting experiments (static compressive tests and cyclic compressive tests) and FEA simulation.

From the experimental results, all fenders are subjected to buckling after the compressive strain is applied. The extent of global buckling of the fender and local buckling at the cylinder ends of the fender was reduced with an increasing wall thickness. From the stress–strain curves obtained from the experiments, the slope of the stress–strain curves changed from negative to positive as the compressive strain increased, and the s-shape characteristic in the stress–strain curves of the five fenders became clearer at thinner wall thicknesses.

From the stress versus strain-energy density curves, the stress response increased with an increasing wall thickness over the considered compressive strain range of 0 to 50%. To quantify the effect of the wall thickness on the energy absorption capacity of the rubber fenders, the energy-effectiveness index $C_{ER}$ was introduced, and it is shown that the fender with the minimal wall thickness (15 mm) achieved the highest energy absorption capacity (i.e., highest $C_{ER}$ value) of the considered fenders. Furthermore, the scale of $R_{D}$ (the ratio of the inner diameter of the hollow-cylinder fender $D_{i}$ to the outer diameter $D_{o}$) had a significant effect on the compression strength and the compression toughness of the current fenders. Both properties were reduced with an increasing value of $R_{D}$ (i.e., a decreasing wall thickness). The results obtained from the OLS regression analyses showed that the compression strength and compression toughness of the fenders could be estimated by using fitted functions of $S_{c,c}\left(\mathrm{MPa}\right)=\;-8.2178\times R_{D}+6.6612$ and $U_{c,c}\left({\mathrm{MJ}/m}^{3}\right)=\;-1.6618\times R_{D}+1.5661$, respectively.

For the cyclic straining tests with a strain range of $\Delta e_{c}=48\%$ in this study, it was found that a clear axial fatigue crack occurred on the surface of the tested sample with wall thickness $T_{k}=25.0\;\mathrm{mm}$ at the loading cycle number of 11,894. In addition, the stress range $\Delta S_{c}$ decreased progressively as the number of cycles increased until 1000 cycles and then remained approximately constant thereafter. Furthermore, when the wall-thickness scale increased, the closed hysteresis loop shifted to higher stress and the closed area within the hysteresis loop also increased.

To conduct the FEA simulation of the buckling of a hollow-cylinder rubber fender, a tensile test on a rubber specimen was conducted to obtain the stress–strain curve, and the hyperelastic Mooney–Rivlin model was used to fit the curve, where the two material coefficients $C_{10}=0.176\;\mathrm{MPa}$ and $C_{01}=0.686\;\mathrm{MPa}$ were obtained, and it was found that the constitutive behavior of the present fenders could be accurately described by using these two material coefficients.

The FEA results show that the absence of a local maximum load in the measured stress–strain curves of the fenders with wall thicknesses of 20–25 mm can be attributed to the rapid formation of a folding-induced self-contact region in the inner surface of the fender during global buckling. The local buckling region shifted toward the center (i.e., mid-height position) of the fender as the wall thickness decreased and resulted in a delay in the development of the folded area on the inner surface. This led to a clearer s-shape characteristic in the stress–strain curve. The FEA results confirm the assertion made in this study that the sensitivity of the s-shape characteristic in the stress–strain curve to the wall-thickness scale is the result of the additional reaction force produced by a folding-induced self-contact phenomenon of the inner fender surface.

Finally, the compression strength and toughness of the present hollow-cylinder rubber fenders can be adequately described not only by the linearly fitted functions $S_{c,c}\left(R_{D}\right)$ and$\;U_{c,c}\left(R_{D}\right)$, respectively, but also by the fifth-degree and the fourth-degree polynomial functions with coefficients determined via experimental fitting.

## Figures and Tables

**Table 1:** Composition of the rubber fenders used in the present study.

Sample Ingredients	Quantity (phr ^1^)
Natural rubber	60.0
Styrene butadiene rubber (SBR)	40.0
Zinc oxide	5.0
Aging agent (4020)	1.5
Aging agent (RD)	1.5
Microcrystalline wax	1.0
Resin	1.5
HAF (high abrasion furnace) carbon blacks	45
Aromatic oil	5.0
Sulfur	2.5
Promoter (DM)	0.65

^1^ phr: parts per hundred of rubber

**Table 2:** Measured mean values of compression strength and compression toughness for five rubber fenders with different wall thicknesses.

$R_{d}=D_{i}/D_{o}$	$S_{c,\;c}\;\left(\mathrm{MPa}\right)$	$U_{c,c}\;\left(\mathrm{MJ}/m^{3}\right)$
0.50	2.5550	0.7227
0.55	2.1528	0.6670
0.60	1.7314	0.5722
0.65	1.2752	0.4762
0.70	0.9388	0.4051

**Table 3:** Polynomial regression coefficients for energy functions $E_{s}\left(e_{c}\right)$ of five rubber fenders.

$T_{k}$	$C_{5}$	$C_{4}$	$C_{3}$	$C_{2}$	$C_{1}$	$C_{O}$
25.0 (mm)	17.5532	−16.8638	3.0513	3.0180	0.1859	−0.0008
22.5 (mm)	12.3199	−9.8528	−0.6615	3.6217	0.1558	−0.0003
20.0 (mm)	14.9751	−11.7837	−1.0947	3.6555	0.1292	−0.0002
17.5 (mm)	13.9804	−9.2136	−3.4634	3.9977	0.0972	0.0000
15.0 (mm)	4.9335	2.9610	−9.2480	4.7736	0.0557	0.0006
